# Analytical sonochemistry: a review

**DOI:** 10.1155/S1463924688000173

**Published:** 1988

**Authors:** P. Linares, F. Lázaro, M. D. Luque de Castro, M. Valcárcel

**Affiliations:** Department of Analytical Chemistry Faculty of Sciences University of Córdoba Córdoba Spain


Journal of Automatic Chemistry, Vol. 10, No. 2 (April–June 1988), pp. 88–94

### Analytical sonochemistry: a review

**P. Linares, F. Lázaro, M. D. Luque de Castro and M. Valcárcel**

*Department of Analytical Chemistry, Faculty of Sciences, University of Córdoba, Córdoba, Spain*

*Analytical chemistry is one of the chemical areas where ultrasonic radiation has been only infrequently used (namely in sample pretreatment, separations, automatic analytical systems and clinical analysis). This auxiliary technique would constitute a powerful aid in the automation of several steps in analytical processes: its advantages and disadvantages, as well as its use and potential in the area of non-invasive detectors, are reviewed in this paper.*

The propagation of ultrasonic waves, characterized by a minimum frequency of 16 kHz, results in rapid fluid movement through compression and rarefaction, the waves generated giving rise to the cavitation phenomenon, i.e. formation and collapse of microbubbles.

The pioneering work on the chemical applications of ultrasound [1] was conducted in the 1920s by Richards and Loomis in their classic survey of the effects of high-frequency sound waves on a variety of solutions, solids and liquids [2]. Despite the diversity of chemical effects of ultrasonic waves discovered by these authors, research conducted since then has been sparse and uneven. In short, the effect of ultrasound is the generation of extreme, punctual temperatures and pressures, which, in general, facilitate and accelerate chemical reactions. Free radicals and ions are generated, chemical layers are dispersed and the contact between the ingredients of the reaction is accelerated. Usually, ultrasonic effects are much more intense in heterogeneous than in homogeneous chemical systems, because emulsification is favoured and mass and heat transfer in two-phase systems is increased [3].

Organic chemistry has been the discipline which has most often used ultrasound to facilitate, improve and accelerate reactions [4–8].

Although analytical chemists have shown little interest in the use of ultrasound, these waves are of great value, their potential at least equalling and generally surpassing that of other conventional methods (distillation, Soxhlet extraction, agitation etc.), which aid the optimum development of some of the steps of an analytical method, such as sample pretreatment, separation, reagent preparation and improvement of reaction yields in continuous systems. Nevertheless, the most important feature of this physical phenomenon are its possibilities for detection, which opens interesting and previously unknown perspectives in analytical control process and, in general, in the sample preparation step for complete automation of analytical process. These and other aspects, as well as their application in clinical chemistry, are dealt with in this paper.

### Sample pretreatment

The most versatile step of the analytical process, sample preparation, has been aided by the use of ultrasound in a variety of forms. Different ways to favour reaction development have been used and compared by a number of researcher groups. Agricultural and environmental chemistry have so far been the areas of applied analysis benefiting to the greatest extent from the use of ultrasound.

Methods for extraction of different analytes from a soil or plant sample are still far from satisfactory and methods based on substantially different principles continue to be proposed with the aim of improving yields and avoiding contamination and alteration of the material. Unfortunately, it seems impossible to fulfil both objectives simultaneously. Another problem arises from the rather long time required to complete the extraction; the time factor plays an important role in the extraction process as regards contamination (and denaturation in the case of organic compounds) in agricultural analysis. Representative examples of the studies conducted in this area are the extraction of humic acids with inorganic reagents from soil [9], in which treatment with pyrophosphate and sonication for 1 h results in yields similar to those achieved after 24 h of mechanical shaking. Nevertheless, subsequent shaking with NaOH gives rise to higher yields which cannot be attained by a larger exposure to ultrasonic radiation. This can be the result of the larger contact time with the chemical reagent in the shaking method. As shown by IR, analysis of analytes extracted by each method have different physical and chemical properties. In the hydrolysis of active principles from plant material (a study of 13 amino-acids) for later identification by paper chromatography [10], it was observed that the procedure involving ultrasound gave higher yields after 5 min exposure than it did with longer exposures of 10 to 15 min; this fact could be the result of degradation increasing with exposure time.

Regarding the extraction of inorganic species, ultrasonic exposure of cations from soil using diluted $HNO_3$ has been studied and compared with conventional shaking methods. The concentration of extracted cations is independent of the intensity of the ultrasonic radiation and dependent upon the reaction temperature and irradiation time. The time needed is always shorter than that required by shaking methods; and the reproducibility of the results depends on the ease with which the temperature can be controlled. Extraction of cationic species from plants by use of HCl and ultrasound, with atomic absorption spectrometry or flame emission detection is more accurate than the dry ashing method [11]. A major improvement in the extraction of phosphate raw materials and P-containing fertilizers is achieved when ultrasound rather than mechanical mixing is used [12].

Other uses of ultrasound in agricultural sample pretreatment include the determination of soil particle size (using ultrasound at 40 $W/cm^2$ for 4 min), which leads to results similar to those obtained by the conventional method. However, in the case of soils including a high proportion of organic matter, $CaCO_3$, or Fe compounds, ultrasound processing causes a decrease in the colloidal clay fraction. Ultrasonic treatment of clay mineral suspensions before deposition on a glass plate is also recommended for obtaining reproducible results, maximum relative reflection intensities and minimum sample preparation time when analysing a large number of samples by X-ray diffraction for qualitative determination of the clay fraction and quantitative evaluation of the mineral components [14]. Several reports of washing plants contaminated by substrate particles have demonstrated how useful ultrasound is in this process [15].

In the environmental area, ultrasonic multielement extraction in atmospheric particulates has been carried out by a simplex-optimized method, with precisions of 10% or less and accuracies of 95% or better in the quantitative extraction of metals [16]. In environmental organic analysis, the extraction of organochloride pesticides from sediments by ultrasonic radiation and shaking provides similar results, with the advantage of a shorter treatment time in the former case [17]. Compared with steam distillation, ultrasonication results in cleaner extractions, albeit with low recoveries. It allows extraction of organochloride pesticides and polychlorinated byphenyls, but not of polynuclear aromatic hydrocarbons. The comparative study performed by Onuska and Terry [18] between extraction of chlorinated benzenes carried out by ultrasounds, Soxhlet extraction and steam distillation at three different levels from different types of bottom sediments showed good features in all cases, although distillation was found to be simpler and more efficient. Likewise, aqueous solutions of trihalomethanes adsorbed onto granular activated carbon were recovered by low-frequency sonication in methanol for 30 min and determined by analysis of methanol extracts using packed column gas chromatography with electron-capture detection. Analytical recoveries averaged 94% [19]. Similar comparative studies of extraction efficiencies for hydrocarbons from sediments polluted by oily drill cuttings were made by Spørstol *et al.* [20], who showed that higher recoveries were obtained by sonic methods.

A comparative study of four extraction techniques (Soxhlet, ultrasonic bath, mechanical shaker and homogenizer-sonicator) applied to munitions residues in soil for later determination by reversed-phase high-performance liquid chromatography was performed by Jenkins and Grant [21]. This demonstrated the usefulness of the ultrasonic bath method, which can be used to automatically process a number of samples simultaneously and is relatively inexpensive. The gentle heating resulting from the sonic bath (≃39 °C) also seems helpful in increasing the rate at which equilibrium is attained.

Ultrasonic radiation facilitates bubble aggregation in samples and release. This effect has been exploited in beer degasification [22]. The concentration of $CO_2$ and $CO_3^=$ in samples can be lowered from 70 to 30 mg/l, and from 23·4 to 0·1 mg/l, respectively, by using a 3 W ultrasound generator producing 30–40 Hz vibrations of 3 μ amplitude [23].

An unusual use of ultrasonication is in dentistry where samples are prepared (dental plaques) for fluorescent micro-assay of the enzymatic activity of lactate dehydrogenase [24].

Organized molecular assemblies are one of the most promising features of future analytical chemistry [25 and 26], among them, vesicles are not systematically applied for analytical purposes, despite them offering interesting possibilities. Sonication is the best method for their formation [27–30].

Thus, ultrasound can compete advantageously with other auxiliary methods of extraction, degassification, homogenization, etc., in sample and also in reagent pretreatment (for example mobile phase degassification in HPLC).

### Use of ultrasound in non-chromatographic separation techniques

The application of ultrasound in separation techniques can be classified according to their chromatographic or non-chromatographic nature.

The almost exclusive use of ultrasound in non-chromatographic separations involves condensed phases, whether solid–liquid (precipitation, ion-exchange), or liquid–liquid (extraction), as no sample preparation with ultrasonic degassification [22 and 23] is included here since the analyte is not isolated from the matrix.

In general, ultrasonic treatment accelerates crystallization rate [31] up to three to eight-fold in the case of calcite, aragonite and sucrose [32]. This results in particles considerably smaller and more uniform in size than those obtained by conventional precipitation. Also, the fractionated precipitation of Th, U and Ce is improved by the use of ultrasound. In fact, a broader precipitation pH range between Th and U is observed when ultrasonic radiation is applied (1·3 as compared to 0·4 with mechanical stirring). This range increases with the radiation intensity and is unaffected by frequency changes; yet, increased frequencies result in flocculation of the precipitated particles [33].

The use of sonication in liquid–liquid extraction results in dramatic emulsification of the two immiscible phases, which enhances the extraction rate [34] and the efficiency of the process to about 50–94% compared to mechanical agitation [35] – the effect decreases with increasing interfacial tension [36].

Ultrasonic radiation improves (96% versus 79%) the regeneration of ion-exchange resins used for eliminating boiler condensates from nuclear reactors [37 and 38]. On the other hand, the ion-exchange [39] and reagent adsorption [40] kinetics are enhanced by ultrasonic treatment in comparison with the undistributed system.

### Use of ultrasound in chromatographic separation techniques

Ultrasound has been used to prepare tuff sorbent for chromatography with increased catalytic and absorption activities [41]. The separation efficiency of liquid chromatography is increased by exposure of the column to ultrasonic vibration applied perpendicularly to the longitudinal axis of the column during the separation [42], probably due to the increased radial dispersion and decreased axial dispersion of the inserted sample bolus [43].

One of the most promising applications of nebulization by ultrasound is the reverse-phase high-performance liquid chromatography/mass spectrometry association, which clearly excels other systems such as direct liquid injection with or without sample preconcentration and evaporation onto a moving belt [44 and 45] owing to the difficulty involved in spraying an aqueous solvent into the vacuum of a mass spectrometer. This feature of ultrasound has been recently exploited for coupling a virtually unmodified gas chromatographic flame photometer as detector for determination of non-volatile analytes by microbore HPLC [46].

### Use of ultrasound in continuous-flow systems

Automatic flow systems have made little use of ultrasonication, and all applications have been aimed at improving methodologies or at on-line detection. Two particular zones of these dynamic configurations are subject to ultrasound: (*a*) that immediately preceding the reaction zone, in order to generate a reagent; (*b*) the transport and reaction zone, as a way of improving the features of the different types of chemical systems used.

(*a*) Sonic chemiluminescence of luminol has been proposed by Yamada *et al.* [47 and 48] for the determination of Co(II) at sub-pg levels by flow-injection analysis, FIA [49], and continuous sample flow [50] methods. The flow diagram designed by these authors consists of a two-channel ($R_1$ and $R_2$) manifold. An aqueous solution containing luminol and sodium hydroxide is supplied through line $R_1$, while $R_2$ denotes a carrier stream for Co(II) and other species, namely an aqueous cobalt(II) solution for the continuous flow method, and pure water or a surfactant solution when Co(II) and other species are injected by means of a 10 μl rotary valve injector (injection method). The streams are delivered by two peristaltic pumps. The alkaline luminol solution is sonicated in a glass sonication cell by means of an exponential horn H. The luminol solution within the sonication cell is kept at a constant volume (*c*. 1·3 ml) during sonication by use of pump tubes with different diameters. In order to ensure adequate and stable cavitation, the sonicated luminol solution is drawn by a third peristaltic pump and mixed with $R_2$ at a Y-joint, to a flow line $R_3$. No oxidant is added. The limit of detection achieved is 0·06 pg (10 μl sample injection) or 0·06 pg/ml (continuous sample flow). The mechanism of the sonic chemiluminescence reaction is different from that of conventional chemiluminescence reactions and ultrasonically generated $O_2$ plays a key role in the Co(II)-catalysed sonochemiluminescence emission.

(*b*) The effect of application of ultrasonic radiation on the transport and reaction zone has only been considered in FIA configurations using both homogeneous and heterogeneous chemical reactions. A previous study of the influence of sonication on physical dispersion of the injected plug was performed. A single-channel FIA configuration using a dye injected into a borax buffer solution was designed for the dispersion study. In general, ultrasound reduces dispersion slightly (increase in the maximum height of the FIA peak by less than 30%). To show the possibilities for using ultrasound in analytical systems involving homogeneous and heterogeneous reactions, different systems previously developed for conventional FIA applications were assayed to critically compare the results obtained in the presence and absence of ultrasound, using the same manifolds and ingredients. From these studies it can be concluded that, in all the systems, the analytical features are more or less significantly enhanced, particularly when interfaces or catalysed reactions are involved. As a rule, the effect of sonication is more marked when detection takes place during the incipient development of reaction on which measurements are based. The application of radiation to the transport and reaction zone of an FIA system has a number of prospective applications of great interest, such as ultratrace determination of catalysts in homogeneous chemical reactions, enhancement of the yield of redox and catalytic reactors, development of continuous liquid–liquid extraction without phase-separation using fluorimetric detection, design of continuous solid(sample)-liquid extractions, of interest to the automatization of procedures (for example the determination of essential elements in soil, metal traces in vegetable material, desorption of pollutants from filtration systems, etc.), all of which show the serious mistake made in ignoring this useful auxiliary tool [43].

The use of ultrasound as a detection system in on-line analysis is explained in the following section.

### Use of ultrasound in and as detection systems

The spraying properties of ultrasound have been used for sample nebulization in ICP–AES for some time [51]. Using ultrasound with conventional aerosol desolvation apparatus makes them preferable to pneumatic nebulizers on account of the smaller and more uniform particle sizes allowing a lower carrier-gas flow-rate to produce an aerosol with greater number density, and the lower sample consumption. Reproducibility is somewhat poorer and the detection limit is one order of magnitude higher. Other advantages are chemical resistance, direct sample introduction, convenient sample change-over and rapid clean-out [52–54]. Similar ultrasonic nebulizers have been coupled for use in graphite furnace-atomic absorption spectrometry [55] and emission spectrography [56], also showing clear advantages over other systems proposed to date.

A German research team has [57–59] developed an apparatus for direct transfer of ultrasonic energy to a reaction solution contained into a photometric cell. A microprocessor controls the process and automates data collection and treatment. The device is used for thermostation of the reacting solution in enzymatic measurements – the inaccuracy of this type of analysis (±20%) in routine clinical laboratory analyses was due to the lack of thermal calibration. A heating rate of 1 °K/s, with an accuracy of 0·01% is achieved; thus, the procedure is 60 times faster and more accurate than thermal conductivity heating.

Nevertheless, the most frequent use of ultrasound in detection systems is energy interaction with the sample and measurement of the change caused in the parameter of interest.

Detection techniques based on this phenomenon use compressional ultrasound (i.e. longitudinal waves) generally in the frequency range 0·5–10 MHz. The power levels are exceedingly low compared with those used for ultrasonic cleaning, emulsification etc. The ultrasound is normally generated by piezo-electric transducers, which can act as transmitters and as receivers. Some form of 'coupling', such as water or grease permits the ultrasound to be transmitted from the transducer to the vessel and vice versa. Some electronic systems can energize the transducers to produce either continuous waves or pulses, detect and amplify received signals, time the echoes and carry out a wide range of data processing activities. Applications are not limited to room temperature – some transducers will tolerate elevated temperature; alternatively, ultrasonic wave guides can be used to allow the transducer to be installed in a cooler part of an industrial plant.

Ultrasonic detectors can be used for identifying the chemical [60] or physical [61] nature of materials, determining the concentration of solutions [62] and mixtures [63], and estimating particle size [64]. It must be admitted, though, that ultrasonic techniques are rarely absolutely specific in the chemical sense. Moreover, they are not normally useful for determining low concentrations.

The parameters involved in ultrasonic detection are velocity, absorption, attenuation, acoustic impedance and scattering.

Velocity, the parameter most widely used [65], has a wide scope of application, namely determination of Pb(II) [62] or Ni(II) [66] by reaction with sodium diethyldithiocarbamate, studies of the molecular mechanism of the phase transition during micelle formation [67] and thermal stability of metal chelates with EDTA [68]; resolution of gas mixtures (for example ethylene in propane saturated with water) [63]; liquid identification; and emulsion concentration [69]. The ultrasonic velocity has even been used as detection system in gas chromatography, where it behaves as a universal detector [70] with a wide dynamic range (six orders of magnitude) [71], but a poor detection limit (in the ng range) [72]. Nevertheless, its use is increasing and it was demonstrated recently that the quantitative determination of a species can be performed with no prior calibration by comparing the response measured in two carrier gases of different molecular weights but equal molar heat capacities [73].

Although ultrasonic absorption measurements are more difficult to make, they have interesting possibilities such as the determination of methanol in water – methanol mixtures [74], the solubility of solid pharmaceuticals [75], the analysis of latexes in polystyrene [60] and the study of metal chelate adducts [76–78].

Ultrasound scattering has frequently been employed for identifying and determining the size of particulates in flowing fluids in semiconductor manufacture [64].

Devices are available for determination of parameters related to the acoustic impedance mismatch between the wall of a vessel and its contents. This, in effect, operates by determining the amount of ultrasound reflected back at the interface between the wall and the liquid. Therefore these devices have the very useful feature of not requiring the liquid to be transparent to ultrasound; this greatly increases the range of materials which they can be used on. At present, they are employed solely to detect whether a liquid is present or absent at a particular point. However, it has been shown that they are sufficiently sensitive to identify liquids and, more speculatively, to measure concentrations of solutions. They also appear to be of use for determining the concentrations of emulsions and, perhaps, solid dispersions, which cannot be analysed ultrasonically by other techniques because they are opaque to ultrasound [69].

### Use of ultrasound in clinical chemistry

Ultrasonication has been used in analyses and diagnoses for the last decade, particularly in medicine. There are few reports of applications of ultrasound to clinical analysis (for example enzyme reactions in biochemistry [79]). Recently, many enzymes have been immobilized on various supports and applied to bioreactors to produce useful compounds. In these systems, the control of the enzyme activity is a very important factor, while the diffusion of the substrate is a limiting factor.

Immobilized reagents have been prepared with the aid of ultrasonic radiation for optimum immobilization. In the case of enzymes, the action of sonication of the enzyme–support mixture favours the physical (adsorption) or chemical (covalent bond) process [80]. Thus, the irradiation of invertase immobilized on Amberlite IRA-94 for 15 min with 200 MHz at 5 °C results in a yield of 90%, higher than in the absence of radiation (75%). The action on catalase immobilized on soil suspensions is much the same. Immobilization of other types of reagent, such as luminol on silica and controlled-pore glass, is also favoured by sonication [81].

Acceleration of substrate diffusion by ultrasonic radiation results in increased α-chymotrypsin activity by a factor between 2 and 2·2 when irradiated with 20 kHz, 10–15 W ultrasonic radiation [82]. This positive effect occurs only when the determining step of the reaction rate is the diffusion of substrate over the support [83].

Another positive effect of ultrasound is the increase of the external surface of supports used for immobilization of α-amylase [84 and 85]. The external characteristics of polymers change significantly under the ultrasonic action through alterations in the superficial structure, as observed by electron-microscopy. After exposure at 22 kHz and 300 W/cm$^2$ for 20 min the external layer of the support is completely eliminated and the material of the porous matrix can attain the external surface. In this way, the specific activity of the immobilized enzyme increases and the apparent Km decreases from 23·8 to 13·7 g/l; yet, it is still seven times higher than that of dissolved amylase.

Activation of immobilized enzymes by ultrasonic radiation has been shown to occur in glucoamylase [86], the activity of which increases by 180% upon irradiation of the enzyme support complex in a $CHCl_3$ solution aimed to remove contaminants.

The effect of ultrasound on the activity of dissolved enzymes is such that therapeutic intensities of continuous waves of 0·88 MHz have no direct influence of the rate of reaction catalysed by creatine–kinase, possibly due to the fact that the first step of an acoustic–biological interaction of ultrasound is the catalytic action on the individual enzyme molecules and this appears to occur at a higher level of organizational complexity [87].

On the other hand, sonication on enzymatic reaction results in denaturation owing to the ultrasound's energy, although the mechanism involved is not yet fully understood. Possible causes cited are the formation of free radicals and hydrogen peroxide giving rise to the oxidation of functional groups at the active enzyme sites. It is frequently noted that these effects occur above cavitation limit, where the acceleration may be as much as 1000 times higher than would correspond to the exciting sound wave [59, 88 and 89].

The ultrasonic energy has been used in immunoassay to favour mass transport through the solid–liquid interface as it can accelerate drastically the antigen-immobilized antibody binding [90]; this is a promising aspect as the rate-limiting step in many solid-phase immunoassays is associated with the slow kinetics of binding macromolecular antigen and conjugate to the immobilized phase. The greater efficiency of ultrasound compared to agitation has been shown in morphine antibodies immobilized on filter paper [91].

### Final remarks

As shown above, ultrasound makes an excellent tool in analytical chemistry, though, surprisingly, has not yet been paid adequate attention.

The different ways in which sample pretreatment can be improved, its beneficial action on separation processes, the incipient but interesting uses in continuous-flow systems as a substantial improvement of the different processes involved and the application in clinical chemistry testify the potential of ultrasound in these areas.

Nevertheless, the greatest potential of the ultrasonic phenomenon is its use as a detection system, as shown in the monograph on ultrasonic methods edited by Alippi and Mayer [61], in evaluation of inhomogeneous material, from the proceedings of NATO Advanced Study Institute of 1985, whose contributors are the most relevant researchers in this area. This prospective use has also been considered by Callis *et al.* in their paper on process analytical chemistry, where the authors consider ultrasound the final, fifth period in the non-invasive era of the analytical process [92].

The CICYT is thanked for financial support (Grant No. PA86-0146).

### References

1. (*a*) Brown, B. and Goodman, J. E., *High intensity Ultrasonics – Industrial Applications* (Van Nostrand, Princeton, 1965); (*b*) Cracknell, A. P., *Ultrasonics* (Wikeham, London, 1980); (*c*) *Ultrasounds: Physical, Chemical and Biological Effects*, Sinclair, F. L., trans. (Consultants Bureau, New York, 1964).

2. Richards, W. T. and Loomis, A. L., *Journal of the American Chemical Society*, **49** (1927), 1497.

3. Bremner, D., *Chemistry in Britain*, July (1986), 633.

4. Boudjouk, P., *Nachrichten aus Chemie, Technik und Laboratorium*, **32**(10) (1983), 798.

5. Toma, S. and Kaliska, V., *Chemicke Listy*, **79**(6) (1985), 578.

6. Lash, T. D. and Berry, D., *Journal of Chemical Education*, **62** (1985), 85.

7. Clough, S., Goldman, E., Williams, S. and George, B., *Journal of Chemical Education*, **63** (1986), 176.

8. Yamawaki, J., Sumi, S., Ando, T. and Hanafusa, T., *Chemistry Letters* (1983), 379.

9. Ramuni, A. U. and Palmiery, F., *Organic Geochemistry*, **8**(4) (1985), 241.

10. Achimescu, V., Georgesan, C. and Cioban, E., *Farmacia*, **30**(3) (1982), 159.

11. Kumina, D. M., Karyakin, A. V. and Gribouskaya, I. F., *Journal of Analytical Chemistry*, **40**(7) (1985), 930.

12. Belyakova, N. I., Preskasch, L. I. and Zaitsev, P. M., *Zhurnal Anatitickeskoi Khimii*, **40**(4) (1985), 648.

13. Ilnicki, P. and Matelska, U., *Rocz Glebozn*, **35**(2) (1984), 15.

14. Shppuntentco, S. A. and Drozdovich, N. I., *Metod Izuch Sostava Sooistv Goru Porod* (1983), 11e

15. McLaughlin, B. E., van Loon, G. W. and Crowder, A. A., *Plant Soil*, **85** (1985) 433.

16. Harper, S. L., Walling, J. F., Holland, D. M. and Pranger, L. J., *Analytical Chemistry*, **57** (1985), 1553.

17. O'Donnell, C., Commission of the European Communities (Rep. EUR 1984, EUR 8518), *Anal. Org. Micropollut. Water*, 36–40.

18. Onuska, F. I. and Terry, K. A., *Analytical Chemistry*, **57** (1985), 801.

19. Alben, K. T. and Kaczmarczyk, *Analytical Chemistry*, **58** (1986), 1817.

20. Sporstol, S., Lichtenthaler, R. G. and Oreld, F., *Analytica Chimica Acta*, **169** (1985), 343.

21. Jenkins, T. F. and Grant, C. L., *Analytical Chemistry*, **59** (1987), 1326.

22. Ruz, J. (private communication).

23. Tebenikhin, E. F., Zhigun, A. M., Shlyapkina, G. N., Samolina, M. A. and Starovoitov, V. S., *Mezhvedomstvennoe Tematicheskii Sbornik Mosk Institut*, **20** (1983) 76.

24. Hanashi, K., Kikuchi, K., Tanaka, H. and Kuwata, F., *Journal of Nihon University School of Dentristy*, **28**(3) (1986), 188.
25. Pelizzetti, E. and Pramauro, E., *Analytica Chimica Acta*, **169** (1985), 1.
26. Love, L. J. C., Hervarte, J. G. and Dorsey, J. G., *Analytical Chemistry*, **56** (1984), 1132A.
27. Fendler, J. H., *Journal Physical Chemistry*, **84** (1980), 1485.
28. Fendler, J. H., *Chemistry in Britain*, Dec. (1984), 1098.
29. Fendler, J. H. and Tundo, P., *Accounts of Chemical Research*, **17** (1984) 3.
30. Rupert, L. A. M., Hockstra, D. and Engberts, J. B. F. N., *Journal of the American Chemical Society*, **107** (1985), 2628.
31. Kardashev, G. A., Salosin, A. V., Shataloc, K. L., Pershina, M. A., Vaganov, V. P. Manukyan, S. G., Mishakin, A. V. and Polyanichev, V. N., USSR. SU, 1,149,992 (CL BO1D9/02) 15 April 1985, Appl. 3, 627, 831, 26 July 1983. From *Otkrytiya Izobret*, 1985 (14) 22.
32. Bogorosh, A. T., Fedotkin, I. M., Gulyi, I. S., Bogorosh, I. S., *Khimicheskaya Tekenologiya* (Kiev), 1984, (2) 42.
33. Kim, Y. S. and Ban, B. C., Taehan. *Kumsok Hakhoe Chi*, **20** (1982), 569.
34. Hoshino, T., Uchiyama, M., and Yukawa, H., *Kagaku Kogaku Ronbunshu*, **10**(3) (1984), 351.
35. Magiera, J. and Tal, B., *Inzynieria Chemicena Procesowa*, **10**(3) (1980), 523.
36. Magiera, J. and Tal, B., *Vergahrenstench*, **14**(10) (1980), 631.
37. Hitachi, Ltd, Spn, Kokai Tokkyo Koho JP 57, 178, 192 (CL, G21F9/12) 02, Nov. 1982, Appl. 81/62, 551, 27 Apr., 1981, 4 pp.
38. Hitachi Ltd, Jpn Kokai Tokkyo Koho JP 59, 150 (84, 150, 547) (CL. B01J49/00) 28 Aug. 1984, Appl. 83/24, 636, 18 Feb. 1983, 5 pp.
39. Cheny, K. L. and Wang, Z., *Mikrochimica Acta*, **2**(5–6) (1982), 399.
40. Medvenev, A. S., Puchkov, V. V., Khasvkii, N. N., Sarukhanov, R. G., Tarasova, I. I. and Evgrafovoa, G. A., *Izvestiya Vysshikh Uchebnykh Zavedenii*, **6** (1985), 51.
41. Torosyan, K. A., USSR, SU, 1,125044 (Cl. B01520/10), 23 Nov., 1984 Appl. 3,540,905, 15 Oct. 1982. From *Otkrytiya, Isobret*, (43) (1984) 34.
42. Aggev, A. N., Zynl'figarov, R. I., Morov, G. P. and Orlov, V. I., USSR. SU, 1081,534 (C.I. G01N31/08), 23 Mar 1984, Appl. 3,548,433, 21 Jan. 1981. From *Otkrytiya, Izofret., Prom. Obraztsy, Tovarnye Znaki*, **11** (1984), 150.
43. Linares, P., Lázaro, F., Luque de Castro, M. D. and Valcárcel, M., *Analytica Chimica Acta*, **200** (1987).
44. Christensen, R. G., White, L., Meiselman, V. S. and Hertz, H. S., *Journal of Chromatography*, **271** (1983), 61.
45. Jeol Ltd, PJN. Kokai Tokkyo Koho, PJ 00, 836 (Cl. H01J49/04) 05 Jan. 1982, Appli. 80/74, 697, 03 Jun. 1980, 3 pp.
46. Karnicky, J. F., Zitelly, L. T. and van der Wal, Sj., *Analytical Chemistry*, **59** (1987), 327.
47. Yamada, M. and Suzuki, S., *Chemistry Letters*, **783** (1983).
48. Komatsu, T., Ohira, M., Yamada, M. and Suzuki, S., *Bulletin of the Chemical Society of Japan*, **59** (1986), 1849.
49. Valcárcel, M. and Luque de Castro, M. D. *Flow Injection Analysis: Principles and Applications* (Ellis Horwood, Chichester, 1987).
50. Goto, M., *Trends in Analytical Chemistry*, **2** (1983) 92.
51. Wendt, R. W. and Fassel, V. A., *Analytical Chemistry*, **37** (1965), 920.
52. Fassel, V. A., Bear, B. R., *Spectrochimica Acta*, Part B, **41B** (1986), 1089.
53. Berman, S. S., McLaren, J. W. and Willie, S. N., *Analytical Chemistry*, **52** (1980), 492.
54. Olson, K. W., Hass, W. J. and Fassel, V. A., *Analytical Chemistry*, **49** (1977) 632.
55. Wennrich, R., Bonitz, U., Brauer, H., Niebergall, J. and Dittrich, K., *Talanta*, **31** (1985), 1035.
56. Aggev, V. S. and Yankouskii, A. A., USSR, SU, 635,788 (Cl. G01N21/00) 07 Oct. 1981, 2,351,231, 26 Apr. 1976. From *Otkrytiya, Izobret, Brom Obraztsy, Fovarnye, Znaki*, **37** (1981), 301.
57. Pradhan, S., Mahesshwari, B. K. and Yadav, R. L., *Indian Journal of Pure and Applied Physics*, **23** (1985), 42.
58. Hagelaner, U., Arnaudov, K. and Faust, V., *Biomedical Technology*, **25** (1980), 242.
59. Hahelauer, U., Faust, U., *Biomedical Technology*, **30** (1985), 264.
60. Gueltepe, M. A., Barret, A., Everett, D. H. and Gueltepe, M. E., *Polymer Colloids*, **313** (1978).
61. Alippi, A. and Mayer, N. G. (Eds), *Ultrasonic Methods in Evaluation of Inhomogenous Materials* (Martinus Nijhoff, 1987).
62. Pradham, S., Maheshwari, B. K. and Yadav, R. L., *Journal of the Indian Chemical Society*, **61** (1984) 619.
63. Tinge, J. T., Mencke, K., Bosgra, L. and Drinkerburg, A. A. H., *Journal of Physics*, **19** (1986), 953.
64. Foote, K. G., U.S., US 4,527,420 (Cl. 73–61; C01N29/100) 09 Jul. 1–85, Appl. 387,741, 11 Jun. 1982; 4 pp.
65. Crouthamel, C. E. and Diehl, H., *Analytical Chemistry*, **20** (1948), 515.
66. Pradham, S., Maheshwari, B. K. and Yadav, R. L., *Indian Journal of Pure and Applied Physics*, **23** (8) (1985), 426.
67. Saidov, A. A., Davidovich, L. A., Nishanov, V. N., Voleisis, A., Karaboev, M. K. and Khabibulloev, J., *Izvestiyn Akademii Nauk Uzbckskoi SSR Seriya Fizika-Matematicheskikh Mauk*, **2** (1984), 59.
68. Sastry, G. L. N., *Indian Journal of Pure and Applied Physics*, **20** (1982), 33.
69. Asher, R. C., *Analytical Proceedings*, **22** (1985), 180.
70. David, D. J., *Gas Chromatographic Detectors* (Wiley, New York, 1974), pp. 144–164.
71. Todd, T. J. and Debord, D., *American Laboratory*, **56** (1970).
72. Hartmann, C. H., *Analytical Chemistry*, **43** (1971), 113A.
73. Skogerboe, K. J. and Yeung, E. S., *Analytical Chemistry*, **56** (1984), 2684.
74. Mukhtarov, R. G. and Ivanov, V. A., *Gazovaya Promyhlehnost*, **2** (1986) 35.
75. Rokhlerko, A. A. and Trukshina, T. S., *Khimiko Farmatsevticheskii Zhurnal*, **19** (1985), 1261.
76. Bartner, J., Rempel, D. and Meloan, C. E., *Analytical Letters*, **13**(A17) (1980), 1513.
77. Chen, C. C. and Petrucci, S., *Journal of Physical Chemistry*, **86** (1982), 2601.
78. Chen, C., Wallace, W., Enring, E. and Petrucci, S., *Journal of Physical Chemistry*, 2541.
79. Chambers, L. A., *Journal of Biological Chemistry*, **117** (1937), 639.
80. Matsushita Electric Works, Ltd, Jpn. Kokai Tokkyo Koho, JP 59 11,184 (8411, 184) (Cl. C12N11/08) 20 Jan. 1984, Appl. 82/120, 012, 10 Jul, 1982, 5 pp.
81. Hool, K. and Nieman, T. A., *Analytical Chemistry*, **59** (1987), 869.
82. Ishimori, Y., Karube, I. and Suzuki, S., *Journal of Molecular Catalysis*, **12** (1981), 253.
83. Schellenberger, A., Schmidt, P., Rosengled, E., Millner, R. and Hansfeld, J., Germ. (East), DD 218,386 (Cl.C12N11/08) 06 Feb. 1985, Appl. 253,352, 25 Jul. 1983, 6 pp.
84. Schmidt, P., Fischer, J., Rosenfeld, E., Millner, R., Haepke, K. and Schellenberger, A., *Angewandtc Makromolekutare Chemic*, **97** (1981), 179.
85. Schmidt, P., Rosenfeld, E. and Fischer, J., *Ultrasound Applications in Medicine and Biology* (New York, 1980).

86. Schmidt, P., Fischer, J., Hettwer, W., Mansfeld, H. W., Wahl, G., Schellenberger, A., Millner, E. and Rosenfeld, E., Germ. (East) DD 150,628 (Cl. C12N13/00) (09 Sept. 1981, Appl. 221.090, 13 May, 1980, 7 pp.
87. Chetverikova, E. P., Pashovkin, T. N., Rozanova, N. A., Sarvazyan, A. P. and Williams, A. R., *Ultrasonics*, **23**(4) (1985), 183.
88. Bergmann, L., *Der Ultraschall* (Hirzel-Verlag, 1954).
89. Lehfeldt, Ultraschall, Würzburg, Vogel-Verlag, 1973.
90. Weng, L., Sizto, N. C., Osorio, B., Hsu, C. J., Rodgers, R. and Litman, D. J., *Clinical Chemistry*, **30** (1984), 1446.
91. Sizo, N. C. and Roux, C. D. G., Euro. Pat. Appl. EP, 137,678 (Cl. G01N33/53) 17 Apr 1985, US Appl. 527,441, 29 Aug. 1983, 12 pp.
92. Callis, J. B., Illman, D. L., and Kowalski, B. R., *Analytical Chemistry*, **59** (1987), 624A.

### CHEMILUMINESCENCE AND FLUORESCENCE IN APPLIED BIOSCIENCES

To be held at the Royal Society of Arts in London, on 10 May 1988, this Conference is a joint event organized by the Automatic Methods Group of the Analytical Division of the Royal Society of Chemistry and the Biotechnology Group of the RSC's Industrial Division. Papers include

General introduction to chemiluminescence and fluorescence, by *J. N. Miller (University of Loughborough)*.

Design and synthesis of fluorescence proteins and the phenomenon of photobleaching, by *R. S. Davidson (The City University, London)*.

Chemiluminescent detection of labels in immunoassay, by *G. Thorpe (Wolfson Research Labs, Queen Elizabeth Medical Centre, Birmingham)*.

Luminescent techniques in the development of rapid, simple, specific and very sensitive assays of macromolecules in complex biological specimens, by *D. Leaback (Coralab, Cambridge)*.

Bioluminescent proteins in cellular and molecular biology, by *A. K. Campbell (University College of Wales, Cardiff)*.

Flow Cytometry – the application of fluorescent probes in the clinical laboratory, by *N. Carter (John Radcliffe Hospital, Oxford)*.

Fluorescent probes for cytosolic ion measurements in living cells, by *T. J. Rink (Smith Kline & French Research Ltd, Welwyn)*.

*Further details can be obtained from Mr S. Langer, Royal Society of Chemistry, Burlington House, London W1V 0BN.*

### NOTES FOR AUTHORS

*Journal of Automatic Chemistry* covers all aspects of automation and mechanization in analytical, clinical and industrial environments. The *Journal* publishes original research papers; short communications on innovations, techniques and instrumentation, or current research in progress; reports on recent commercial developments; and meeting reports, book reviews and information on forthcoming events. All research papers are refereed.

### Manuscripts

Two copies of articles should be submitted. All articles should be typed in double spacing with ample margins, on one side of the paper only. The following items should be sent: (1) a title-page including a brief and informative title, avoiding the word 'new' and its synonyms; a full list of authors with their affiliations and full addresses; (2) an abstract of about 250 words; (3) the main text; (4) appendices (if any); (5) references; (6) tables, each table on a separate sheet and accompanied by a caption; (7) illustrations (diagrams, drawings and photographs) numbered in a single sequence from 1 upwards and with the author's name on the back of every illustration; captions to illustrations should be typed on a separate sheet. Papers are accepted for publication on condition that they have been submitted only to this *Journal.*

### References

References should be indicated in the text by numbers following the author's name, i.e. Skeggs [6]. In the reference section they should be arranged thus:

to a journal

Manks, D. P., *Journal of Automatic Chemistry*, **3** (1981), 119.

to a book

Malmstadt, H. V., in *Topics in Automatic Chemistry*, Ed. Stockwell, P. B. and Foreman, J. K. (Horwood, Chichester, 1978), p. 68.

### Illustrations

Original copies of diagrams and drawings should be supplied, and should be drawn to be suitable for reduction to the page or column width of the *Journal*, i.e. to 85 mm or 179 mm, with special attention to lettering size. Photographs may be sent as glossy prints or as negatives.

### Proofs and offprints

The principal or corresponding author will be sent proofs for checking and will receive 50 offprints free of charge. Additional offprints may be ordered on a form which accompanies the proofs.

*Manuscripts should be sent to either Dr P. B. Stockwell or Ms M. R. Stewart, see inside front cover.*

